# Effect of Native Defects on Transport Properties in Non-Stoichiometric CoSb_3_

**DOI:** 10.3390/ma10030287

**Published:** 2017-03-14

**Authors:** Paula R. Realyvázquez-Guevara, Francisco J. Rivera-Gómez, Alejandro Faudoa-Arzate, María E. Botello-Zubiate, Renee J. Sáenz-Hernández, Carlos R. Santillán-Rodríguez, José A. Matutes-Aquino

**Affiliations:** Centro de Investigación en Materiales Avanzados, S.C., Av. Miguel de Cervantes 120, Complejo Industrial Chihuahua, Chihuahua 31136, Mexico; paula.realyvazquez@cimav.edu.mx (P.R.R.-G.); francisco.rivera@cimav.edu.mx (F.J.R.-G.); alejandro.faudoa@cimav.edu.mx (A.F.-A.); eugenia.botello@cimav.edu.mx (M.E.B.-Z.); joselin.saenz@cimav.edu.mx (R.J.S.-H.); carlos.santillan@cimav.edu.mx (C.R.S.-R.)

**Keywords:** thermoelectrics, skutterudites, low temperature, electrical transport, native defects

## Abstract

The effect of native defects originated by a non-stoichiometric variation of composition in CoSb_3_ on I-V curves and Hall effect was investigated. Hysteretic and a non-linear behavior of the  I-V curves at cryogenic temperatures were observed; the non-linear behavior originated from the Poole-Frenkel effect, a field-dependent ionization mechanism that lowers Coulomb barriers and increases emission of charge carriers, and the hysteresis was attributed to the drastic decrease of specific heat which produces Joule heating at cryogenic temperatures. CoSb_3_ is a narrow gap semiconductor and slight variation in the synthesis process can lead to either *n*- or *p*-type conduction. The Sb-deficient CoSb_3_ presented an *n*-type conduction. Using a single parabolic model and assuming only acoustic-phonon scattering the charge transport properties were calculated at 300 K. From this model, a carrier concentration of 1.18 × 10^18^ cm^−3^ and a Hall factor of 1.18 were calculated. The low mobility of charge carriers, 19.11 cm^2^/V·s, and the high effective mass of the electrons, 0.66 *m*_0_, caused a high resistivity value of 2.75 × 10^−3^ Ω·m. The calculated Lorenz factor was 1.50 × 10^−8^ V^2^/K^2^, which represents a decrease of 38% over the degenerate limit value (2.44 × 10^−8^ V^2^/K^2^).

## 1. Introduction

Thermoelectric (TE) materials can generate electric potentials when subjected to a temperature gradient (Seebeck effect) and, conversely, they can transfer heat against a temperature gradient when a current is applied against the generated potential (Peltier effect) [1]. TE materials have attracted great research interest during the past decades due to their potential applications for power generation and are considered as an attractive option for waste heat recovery systems. The main obstacle to widespread use of thermoelectric TE materials is their low efficiency for converting thermal energy into electric energy [2]. The performance of a TE material is related to the dimensionless thermoelectric figure of merit, *ZT*, defined as *ZT* = *S*^2^σ*T*/(κ_e_ + κ_L_), where *S* is the Seebeck coefficient, *T* is the absolute temperature, σ is the electrical conductivity, κ_e_ and κ_L_ are the electronic and lattice thermal conductivities, respectively. The higher the *ZT*, the greater the TE conversion efficiency. But *S*, σ and κ_e_ are entangled, thus, improvement of one parameter usually adversely influences the others [3]. Therefore, high *ZT* values arise from the beneficial combination of high power factor, *S*^2^σ, and low thermal conductivity, (κ_e_ + κ_L_) [4]. Various approaches have been taken to improve the power factor, such as band engineering and electronic state distortion to enhance the Seebeck coefficients [5,6]. Thermal conductivity can be lowered by either filling the voids of the crystal structure in TE materials with cage-like structures like skutterudites [7] or by nanopatterning and nanostructuring [8,9].

Skutterudites are good examples of the phonon-glass-electron-crystal (PGEC) concept proposed by Slack [4] in 1995 and, therefore, they are regarded as promising candidates for next-generation TE materials for electrical power generation using either solar energy or waste heat [10]. The general formula of skutterudite compound is *AB*_3_, where *A* is a VIIIB group transition metal such as Co, Ir or Rh and *B* is a pnictogen atom such as Ar, P, or Sb. The crystal structure of these compounds belongs to the space group Im$\bar{3}$ and the unit cell consists of 32 atoms, where six out of the eight simple cubic metal subunits are filled with a near square planar ring formed by four pnictogen atoms (Sb_4_).

Deviation from stoichiometry in skutterudites is a common issue that can result in a large variation in electrical transport properties (i.e., Seebeck coefficient and electrical conductivity) [11]. CoSb_3_ has been reported with either *p*- or *n*-type conduction [10,11,12]. In Sb-rich compositions, a *p*-type conduction has been observed; while in Co-rich compositions, there is a transition from *n*- to *p*-type conduction above 500 K. Understanding and controlling the native defects originated in non-stoichiometric undoped CoSb_3_ is essential for the optimization of TE properties of skutterudites.

The influence of Sb deficiency on electrical transport properties was investigated. The temperature dependence of the I-V characteristics allowed to understand different aspects of the current-transport mechanisms. A shift from linear to non-linear and hysteretic behavior was observed at low temperatures. The non-linear behavior is interpreted on the basis of the appearance of field-dependent mechanisms, which are normally too weak at high temperature. The hysteresis was attributed to the drastic decrease of specific heat at low temperatures which produces Joule heating at cryogenic temperatures. Finally, some charge transport coefficients were calculated in the Sb-deficient sample at 300 K within the framework of a single parabolic band approximation with a dominant carrier scattering by acoustic phonons. CoSb_3_ is a narrow-gap semiconductor, and slight variations in non-stoichiometric composition can result in large variations in charge transport properties. In this work, the focus has been to study the influence of native defects on charge transport properties.

## 2. Experimental Procedures

Polycrystalline skutterudite (CoSb_3_) samples were prepared using a sequence of processes, including arc-melting, melt spinning, grinding, cold pressing, and annealing. To avoid oxidation, each individual process was carried out under a protective Ar atmosphere. Co pieces with a purity of 99.5% (Alfa Aesar, Tewksbury, MA, USA) and Sb shoot with a purity of 99.9999% (Alfa Aesar) were used as raw materials. Sb has a low vapor pressure, thus volatilization of Sb during the fabrication of CoSb_3_ was compensated by adding 10 wt % in excess to the stoichiometric amount of Sb. As a first step, an arc melting furnace was used to obtain an ingot. Afterwards, the bulk sample was re-melted under vacuum in an induction furnace and the melted material was ejected onto the copper wheel of a melt-spinning system, rotating with a tangential velocity of 30 m/s. The resulting product was a mix of finely divided ribbon flakes and wider ribbon sections. The ribbons were grounded using an agate mortar and the resulting powder was loaded into a die cavity of 8 mm × 8 mm. Then, the powder was cold pressed under a uniaxial pressure of 5 MPa during 1.5 min. Finally, the pellet was annealed at 650 °C for 2 h with heating and cooling rates of 10 °C/min.

X-ray diffraction (XRD) phase identification was carried out using a PANalytical X’Pert PRO (PANalytical, Almelo, The Netherlands) with Cu-Kα radiation. Rietveld refinement was performed using the Fullprof program [13].

The morphology of the ribbons and of the annealed sample were analyzed by scanning electron microscopy (SEM) (JEOL-JSM 5800, JEOL, Tokyo, Japan). Backscattered electron imaging (BEI) and secondary electron imaging (SEI) were employed to observe the details of the microstructure. Chemical composition and sample homogeneity were checked by energy-dispersive X-ray spectrometry (EDS, Tokyo, Japan) microanalysis attached to the SEM. The actual composition was determined by averaging the distinct zones on the surface of each sample and by normalizing the resulting chemical formula to one cobalt atom.

I-V curves and Hall effect of the annealed prism-shaped sample (8 × 8 × 2 mm^3^) were measured by the AC four-probe method between 2 and 300 K. The sample was soldered to the puck of the AC transport option of a physical property measurement system (PPMS, Quantum Design, San Diego, CA, USA) using four silver wires and Pb/Sn braze.

Electrical conductivity, thermal conductivity and Seebeck coefficient were measured between 3 and 400 K on 14 × 2 × 2 mm^3^ bar samples using the thermal transport option (TTO) of a physical property measurement system (PPMS, Quantum Design). The copper leads were attached to the sample using a two-component silver epoxy (EPOTEK, Billerica, MA, USA). The leads and the epoxy were both provided by Quantum Design.

## 3. Results and Discussion

### 3.1. Microstructure

**Figure 1:**
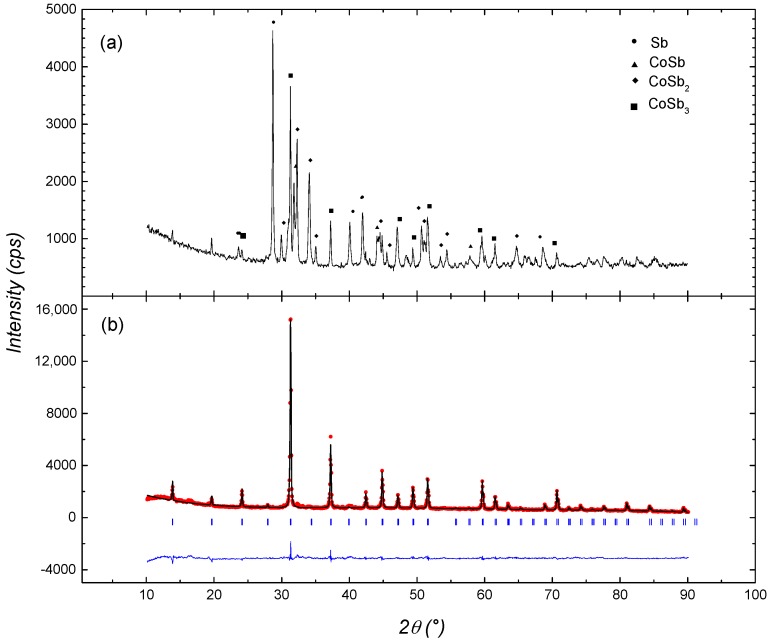
(**a**) XRD pattern of the ribbons obtained by melt spinning; and (**b**) Rietveld refinement of the XRD pattern of the annealed bulk sample.

The X-ray diffraction (XRD) powder pattern of ribbons prepared by melt spinning, is shown in Figure 1a. A complex phase composition with Sb, CoSb, CoSb_2_, and CoSb_3_ phases were detected, with Sb as the main phase.

Figure 1b shows the Rietveld refinement using the FullProf program for the X-ray diffraction pattern of the annealed bulk sample, where a single-phase sample with a lattice parameter equal to 9.0302 Å was determined.

**Figure 2:**
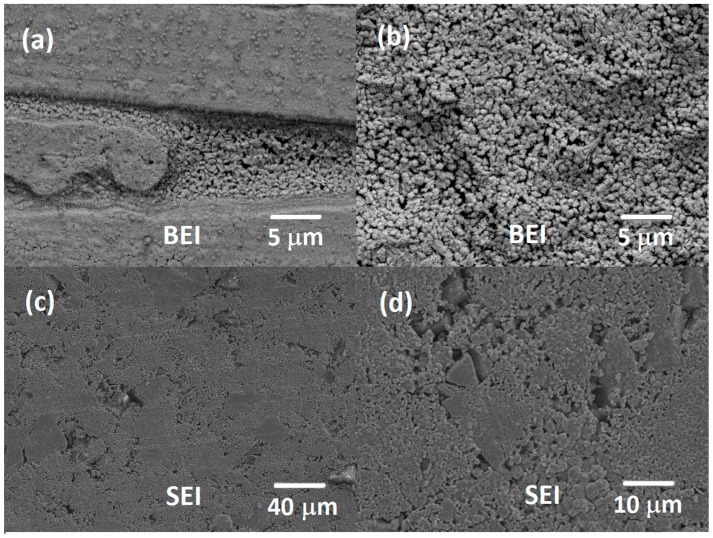
(**a**) BEI of the contact surface of the ribbon and (**b**) BEI of the free surface of the ribbon; (**c**) SEI of the annealed bulk material at 500× and (**d**) 5000×.

The melt-spun ribbons have two distinct surfaces: one that directly touches the copper wheel (contact surface) and another which does not contact the copper wheel (free surface). These two sides differ in their microstructure. The microstructure on the contact surface exhibit two different zones, one of them with rounded grains located into depressions with size distribution between 0.5 and 1.25 µm, and other zone with a mix of rounded, square and irregular grain shapes, with a broader size distribution, from 0.1 to 1 µm, as observed in SEM-BEI image in Figure 2a. The free surface exhibits rounded grains with well-defined grain boundaries, as shown in SEM-BEI images in Figure 2b, with a grain size distribution between 0.85 and 1.5 µm. These differences in the microstructures of both surfaces were to be expected, since the contacting ribbon surface has a higher cooling rate.

The annealing of the grounded ribbons resulted in a growth of the grain size, obtaining grains from 0.6 to 50 µm, as shown in Figure 2c,d.

The elemental composition was determined by averaging 10 separate elemental analyses performed at random zones of the sample. The average atomic percentage of Co was normalized to 1 since it is the least likely element to be lost during processing. The average stoichiometric ratio of Sb was derived from this normalized value. A nominal composition of CoSb_2.81_ was determined, which indicates a Sb-deficiency. This deviation from the stoichiometric relation is a very common issue encountered during the fabrication of CoSb_3_ [14]. The Sb-deficiency provides extrinsic carrier that have impact on the band gap and, hence, on electronic transport properties.

### 3.2. Electric Transport Properties

**Figure 3:**
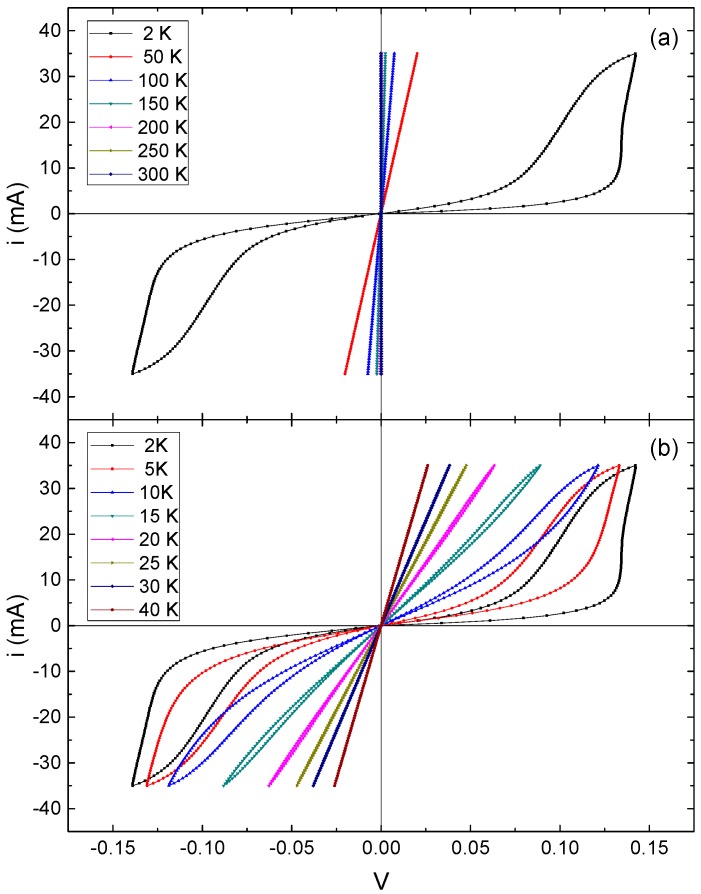
(**a**) Current-voltage (I-V) characteristics between 2 and 300 K, at *T* = 2 K a non-linear and hysteretic behavior appears; and (**b**) a closer temperature interval, from 2 and 40 K, showed that the non-linear and hysteretic behavior appears at *T* < 20 K.

Figure 3a shows the current-voltage (I-V) characteristics from 2 to 300 K. The I-V curve measured at 2 K shows a non-linear and hysteretic behavior. I-V measurements taken at closer temperature intervals, between 2 to 40 K, are shown in Figure 3b. Above *T* = 20 K, the I-V curves are linear, indicating that the resistance is a constant at a given temperature. In other words, the current (I) is proportional to the potential difference (V), resulting in ohmic-like behavior. As the temperature decreases below *T* = 20 K a non-linear and hysteretic behavior appears. This behavior becomes more significant as the temperature decreases.

At cryogenic temperatures, electrons are generally trapped in localized states, and they do not have enough energy to get out of its localized state. Although the temperature reduction causes a suppression of the thermal excitation mechanism, which promote electrons from the valence band into the conduction band, the field-dependent ionization mechanism, which is usually too weak at 300 K to be observed, become important at lower temperatures. Generally speaking, the application of strong electric fields enhances emission of carriers due to Poole-Frenkel effect, phonon-assisted tunneling, and direct tunneling. The experimental investigations of carrier emission applying low-electric fields, below 1 kV/cm, which is our case, has been attributed to the Poole-Frenkel effect, which is caused by the lowering of the Coulomb barrier [15].

As shown in Figure 3b, as temperature decreases and a non-linear behavior appears, there is a critical electric field beyond which the electric current increases more rapidly than in the linear regime. The voltage at which the electric conduction increases more rapidly is known as breakdown voltage and the magnitude of the breakdown voltage in which field-dependent ionization mechanism appears increase as temperature decreases. The effects discussed above disappears at *T* > 20 K, where thermal energy starts to promote carriers from the valence band into the conduction band (ohmic-like behavior).

Finally, it should be mentioned that, for sufficiently large currents, self-heating of the sample can occur at cryogenic temperatures [16]. This self-heating results from the fact that the specific heat decreases as the temperature is lowered till, as proposed in the Debye model, it approaches to zero at absolute zero. As a result, the lattice temperature may become much higher than the ambient temperature due to Joule heating. This heating changes the charge carrier concentration in the semiconductor, given rise to the hysteretic behavior of the I-V curves.

**Figure 4:**
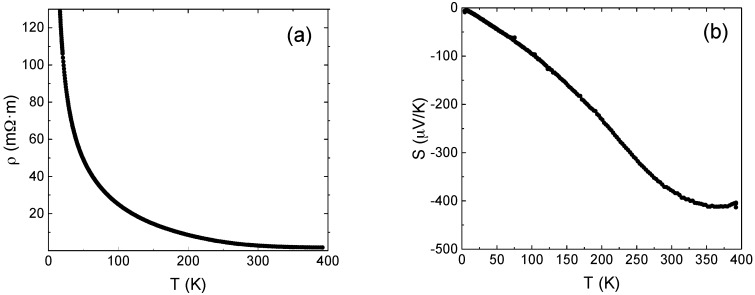
Temperature dependence between 3 and 400 K of (**a**) electrical resistivity and (**b**) Seebeck coefficient.

The temperature dependence of the electrical resistivity of CoSb_2.81_, Figure 4a, shows that ρ decreases with increasing temperature, which is the classical semiconductor behavior. A value of 2.7 mΩ·m was observed in the sample at 300 K which is similar to the value reported by other authors [17]. The reported values for CoSb_3_ resistivity at room temperature differ by three orders of magnitude, as reported by Furuyama et al. [18], from 10^−1^ to 10^−4^ Ω·m. Samples with Sb-deficiency tends to present higher electrical resistivity, due to changes on the band structure caused by point variations of non-stoichiometry and defect structure [11].

Variations of non-stoichiometry in CoSb_3_ can lead to either *n*-type or *p*-type conduction [19,20]. Sb-enriched samples have *p*-type conduction and Sb-deficient samples will lead to an excess of electrons in the conduction band, showing an *n*-type behavior [21]. Although Sb-vacancy has been tentatively considered to be an important native point defect, density-functional theory calculations performed by Park et al. [14] determined that in Sb-deficient samples, interstitial Co ($Co_{i}$) is the most stable isolated native point defect. According to Park et al., $Co_{i}\;$is an acceptor-like defect, with a very low formation energy (0.5 eV). This low value can yield a high concentration of $Co_{i}$ and the formation of a$\;Co_{i}$-pair, which is a stable defect at low temperature. The $Co_{i}$-pair is a donor-like defect. However, recent calculations performed by Li et al. [22] determined that a $Co_{i}$-pair is not expected to form during the process and the breakage of the Sb_4_ rings induced by $Co_{i}$ or a $Co_{i}$-pair promote the formation of lone pairs of electrons in Sb.

The temperature dependent values of the Seebeck coefficient, *S*, showed in Figure 4b, are negative in the measuring temperature interval, meaning an *n*-type conduction. In CoSb_2.81_, the non-stoichiometry variation provides extra electrons. The absolute value of *S* increases up to around 364 K, reaching a maximum of 412 μV/K. This value is in good agreement with the value obtained by W.S. Liu et al. [23]. This high obtained absolute value is originated by a lower charge carrier density originated by non-stoichiometry. The typical reported values for the Seebeck coefficient varies from ~+100 to −400 μV/K, at room temperature [23]. It can be observed that the absolute Seebeck coefficient starts to decrease at temperatures above 364 K, which is consistent with the observation made by other authors in *n*-type CoSb_3_ [11]. The change of the slope is caused by the transition from weak extrinsic conduction, originated by the Sb-deficiency, to the intrinsic conduction.

Goldsmind and Sharp developed the analytical expression $E_{g}=2e{\left|S\right|}_{max}T_{max}$ relating the bandgap, $E_{g}$, the absolute maximum total Seebeck coefficient, ${\left|S\right|}_{max}$, and the temperature at which it occurs, $T_{max}$. From the measured absolute maximum Seebeck coefficient, the calculated value for $E_{g}$ is 0.48 eV, which is comparable with the values reported by Wei-Shu et al. [11]. Experimental observations made by Wei-Shu et al. led to the conclusion that *n*-type CoSb_3_ has a larger energy bandgap than *p*-type CoSb_3_. According to the defect state model proposed by Park et al. [14], the highest 3d-like state of *Co_i_* pairs becomes deeper inside the bandgap and, thus, the energy separation between the valence-band maximum and conduction-band minimum becomes higher, increasing the value of the band gap in *n*-type CoSb_3_.

According to band structure calculations for CoSb_3_, the valance band has the first maxima at the Г point and has 12 equivalent second maxima along the ГN in the k space. On the other hand, the conduction band has the first minimum at the Г point and has 12 equivalent second minima along the ГN. This structure can be assumed as two valance bands and two conduction bands [24]. One of the valence bands shows a strong non-parabolicity and the scattering of charge carriers are due to non-polar optical phonons, polar optical phonons, ionized impurities, and intervalley transitions. In the remaining bands (one valance band and the two conduction bands) a parabolic dispersion relation is assumed. This parabolic bands are dominated by acoustic-phonon scattering. In a stoichiometric 1:3 compound, it is expected that both, the valance band and the conduction band, contribute with charge carriers. However, the non-stoichiometry of the sample provides extra free electrons, obtaining some extrinsic nature in the sample, where the conduction is primary due to the conduction band at 300 K.

From the experimental Seebeck coefficient value obtained at 300 K, *S* = −383 µV/K, the reduced Fermi level $\eta =-(E_{C}-E_{F})/k_{B}T$ can be numerically calculated assuming a single-parabolic conduction band:

(1) $$S=-\frac{k_{B}}{e}\left[\frac{\left(2+\lambda \right)F_{1+\lambda }\left(\eta \right)}{\left(1+\lambda \right)F_{\lambda }\left(\eta \right)}-\eta \right]\;$$

where $k_{B}$ is the Boltzmann constant, e is the elementary charge, η is the reduced Fermi level, λ is a parameter that reflects the dominant scattering mechanism of charge carriers, h the Plank constant, $F_{i}$ the Fermi integral of order i:

(2) $$F_{i}=\int_{0}^{\infty }\frac{x_{i}}{e^{\left(x-\eta \right)}+1}dx$$

Assuming that λ=0 for acoustic-phonon scattering which is the generally accepted dominant scattering mechanism for the conduction parabolic band [25,26], the calculated reduced Fermi level was −2.3965, which means that the Fermi level lies in the bandgap, a characteristic in non-degenerated semiconductors.

Usually, the Hall factor *r_H_* is assumed to be equal to 1, this assumption is valid only in degenerated systems or for energy-independent carrier relaxation time. However, is expected that this factor value will be different from 1 in non-degenerated semiconductors. The value of *r_H_* can be estimated by:

(3) $$r_{H}=\frac{3}{2}F_{\frac{1}{2}}\left(\eta \right)\left[\frac{\left(\frac{1}{2}+2\lambda \right)F_{2\lambda -\frac{1}{2}}\left(\eta \right)}{{\left(1+\lambda \right)}^{2}F_{\lambda }^{2}\left(\eta \right)}\right]$$

assuming again that λ=0 and taking the previously calculated value of η, $r_{H}=1.17$. In non-degenerated semiconductors, $r_{H}$ can vary from 1.18 for scattering by acoustic phonons up to 1.93 for scattering by ionized impurities [27]. The calculated value is really close to 1.18.

**Figure 5:**
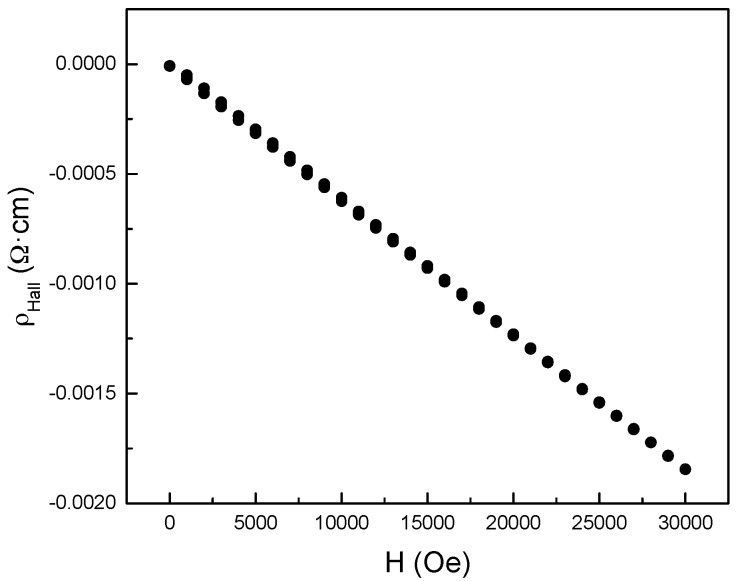
Hall resistivity as a function of magnetic field taken at *T* = 300 K. At this temperature, the linear magnetic field dependence indicates a single type of charge carrier.

The negative value of Hall coefficient, *R_h_* = −6.1590 cm^3^/C, determined from the slope of Hall resistivity variation with magnetic field, from −3 to 3 T, as shown in Figure 5, confirmed that the sample has a *n*-type conduction at 300 K. The linear field dependence of the Hall resistivity corresponds to that of a single type of charge carrier.

In a parabolic dispersion relation, the carrier concentration can be obtained by:

(4) $$n=\pm \frac{r_{H}}{R_{H}e}$$

where e is the elementary charge and $r_{H}$ is the Hall factor. Taking the previously calculated value for $r_{H}$ equal to 1.17, the carrier concentration, calculated from the measured Hall coefficient, was 1.19 × 10^18^ cm^−3^.

Combining η obtained from Seebeck coefficient fitting at 300 K and n, the effective mass of the electron $m^{*}$ can be estimated assuming a parabolic band where acoustic phonon scattering dominates by:

(5) $$n=4\pi {\left(\frac{2m^{*}k_{B}T}{h^{2}}\right)}^{3/2}F_{1/2}\left(\eta \right)$$

obtaining a value of $0.60\;m_{0}$, where $m_{0}$ is the free electron mass. Skutterudites are a kind of compound where the hole effective mass is significantly smaller than the electron effective mass [28].

Taking the value of the resistivity as 2.75 × 10^−3^ Ω·m at 300 K, Hall mobility $\mu_{H}=R_{H}/\rho$ presented a value of 16.30 cm^2^/V·s at 300 K. *N*-type CoSb_3_ mobility are substantially lower compared with the reported values of *p*-type CoSb_3_ [29], resulting in higher electrical resistivity values.

The Lorenz factor was calculated by the formula:

(6) $$L=\frac{k_{B}^{2}}{e^{2}}\frac{\left(1+\lambda \right)\left(3+\lambda \right)F_{\lambda }\left(\eta \right)F_{2+\lambda }\left(\eta \right)-{\left(2+\lambda \right)}^{2}F_{1+\lambda }{\left(\eta \right)}^{2}}{{\left(1+\lambda \right)}^{2}F_{\lambda }{\left(\eta \right)}^{2}}$$

using λ=0 and the previously calculated η value, a Lorenz factor of 1.50 × 10^−8^ V^2^/K^2^ was obtained. It is important to note that the Lorenz number, as described by the free-electron model, is not an accurate value for most materials and in a given material depends on the detailed band structure, position of the Fermi level and the temperature [30]. The obtained value is significantly lower than the value of the degenerated electron gas, $L_{0}=2.44\times 10^{-8}\;V/K^{2}$. In non-degenerated semiconductors, the Lorenz factor decreases up to 40% of the value of the free electron model [31,32].

### 3.3. Thermal Transport Properties

**Figure 6:**
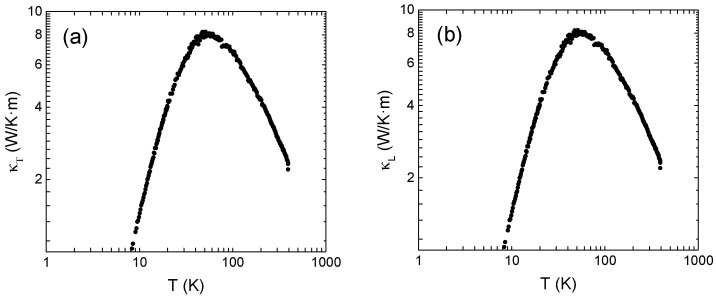
(**a**) Thermal conductivity and (**b**) lattice thermal conductivity for CoSb_2.81_.

As shown in Figure 6a, the total thermal conductivity presents a maximum value of 8.24 W/K·m at 50 K. For temperatures lower than the observed maximum, thermal conductivity decreases due to the decreasing of specific heat when the temperature decreases, as proposed in the Debye model. For temperatures higher than the maximum thermal conductivity decreases due to thermally-activated phonon-phonon Umklapp scattering process. The lattice thermal conductivity, $\kappa_{L}$, was obtained by subtracting the electronic contribution, $\kappa_{e}=L_{0}T/\rho$, from the total thermal conductivity, $\kappa_{T}$, where the assumed value for the Lorenz number was the previously calculated one $L_{0}=1.50\times 10^{-8}\;V/K^{2}$. The room-temperature lattice thermal conductivity for CoSb_2.81_ was 3 W/K·m for, which is one of the lowest measured values for this compound and is comparable with the low conductivity obtained by high synthesis pressure [33]. As proposed in the Debye-Callaway model [34], the phonon scattering process can be represented by a relaxation time and this time is derived from a combination of relaxation times for scattering by defects, phonon-phonon scattering, boundary scattering, and resonant scattering. The sample is not nanostructured and, thus, the low thermal conductivity in CoSb_2.81_ observed at room temperature could be related to the scattering of phonons by point defects. This reduction on the lattice thermal conductivity by the presence of defects was predicted by Liu et al. [35].

### 3.4. Figure of Merit

**Figure 7:**
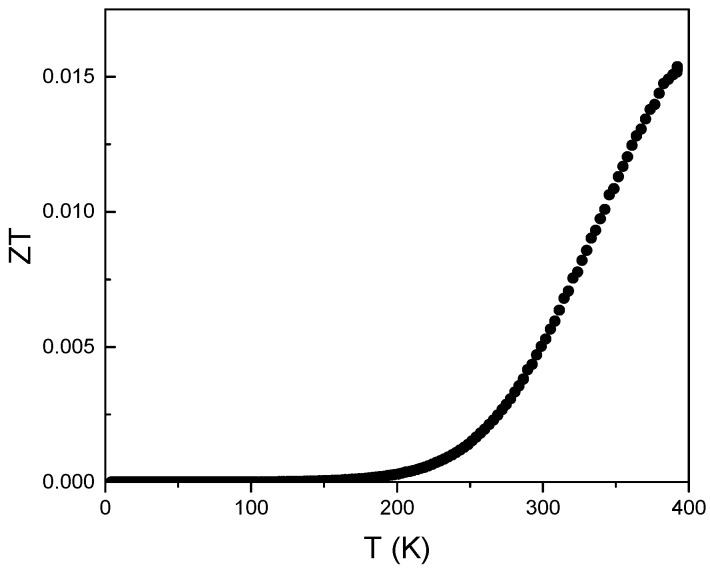
Temperature dependence of the figure of merit between 2 and 400 K.

Figure 7 shows the temperature dependence of the Figure of Merit, *ZT*, from 3 to 400 K, where a value of *ZT* = 0.015 was calculated at the maximum measuring temperature. This value is comparable to other reports measured at the same temperature [34]. The presence of native defects affected the electrical transport, however, restrain the thermal transport.

## 4. Conclusions

A non-linear and a hysteretic behavior in the I-V curves at low temperature were observed. The non-linear behavior was originated from the Poole-Frenkel effect, a field-dependent ionization mechanism, which lowers the Coulomb barrier enhancing the emission of charge carriers. The hysteresis for *T* < 20 K was attributed to the drastic decrease of specific heat which produces Joule heating at cryogenic temperature. The *n*-type electrical conduction of the Sb-deficient sample at 300 K has been attributed to the non-stoichiometry. Within the single parabolic model, and assuming acoustic-phonon scattering, which is the generally accepted dominant scattering mechanism in filled and unfilled skutterudites, some charge transport parameters were calculated from the Seebeck coefficient and Hall measurements performed at 300 K. The numerically calculated reduced Fermi level was estimated to be −2.3965, which means that the Fermi level lies into the band gap, a characteristic in non-degenerated semiconductors. The carrier concentration was about 1.18 × 10^18^ cm^−3^. Hall factor, which usually is assumed to be 1, was calculated to be 1.17. The Hall mobility of the charge carriers was small and estimated to be 19.11 cm^2^/V·s caused by the heavy effective mass of the electrons, 0.66 *m*_0_. This low mobility and high effective mass adversely affected the electrical resistivity, obtaining a high resistivity value, 2.75 × 10^−3^ Ω·m, measured at 300 K. Finally, a calculated value of the Lorenz factor of 1.50 × 10^−8^ V^2^/K^2^, usually this factor is assumed to be 2.44 × 10^−8^ V^2^/K^2^ in degenerated semiconductor, however, in non-degenerated semiconductors it depends on the detailed band structure position of the Fermi level and the temperature. The presence of native defects in the non-stoichiometric sample adversely affected the electrical transport properties, however, the thermal conductivity was drastically reduced due phonon scattering by point defects.
